# Atrial fibrillation, antithrombotic treatment, and cognitive aging: A population-based study

**DOI:** 10.1212/WNL.0000000000006456

**Published:** 2018-11-06

**Authors:** Mozhu Ding, Laura Fratiglioni, Kristina Johnell, Giola Santoni, Johan Fastbom, Petter Ljungman, Alessandra Marengoni, Chengxuan Qiu

**Affiliations:** From the Aging Research Center (M.D., L.F., K.J., G.S., J.F., A.M., C.Q.), Department of Neurobiology, Care Sciences and Society, Karolinska Institutet and Stockholm University; Stockholm Gerontology Research Center (L.F.), Stockholm; Institute of Environmental Medicine (P.L.), Karolinska Institutet, Stockholm; Department of Cardiology (P.L.), Danderyd Hospital, Stockholm, Sweden; and Department of Clinical and Experimental Sciences (A.M.), University of Brescia, Italy.

## Abstract

**Objective:**

To examine the association of atrial fibrillation (AF) with cognitive decline and dementia in old age, and to explore the cognitive benefit of antithrombotic treatment in patients with AF.

**Methods:**

This population-based cohort study included 2,685 dementia-free participants from the Swedish National Study on Aging and Care in Kungsholmen, who were regularly examined from 2001–2004 to 2010–2013. AF was ascertained from clinical examination, ECG, and patient registry. Global cognitive function was assessed using the Mini-Mental State Examination. We followed the DSM-IV criteria for the diagnosis of dementia, the NINDS-AIREN (National Institute of Neurological Disorders and Stroke and Association Internationale pour la Recherché et l'Enseignement en Neurosciences) criteria for vascular dementia, and the NINCDS-ADRDA (National Institute of Neurological and Communicative Disorders and Stroke and the Alzheimer's Disease and Related Disorders Association) criteria for Alzheimer disease. Data were analyzed using multiple linear mixed-effects and Cox regression models.

**Results:**

We identified 243 participants (9.1%) with AF at baseline. During the 9-year follow-up period, 279 participants (11.4%) developed AF and 399 (14.9%) developed dementia. As a time-varying variable, AF was significantly associated with a faster annual Mini-Mental State Examination decline (β coefficient = −0.24, 95% confidence interval [CI]: −0.31 to −0.16) and an increased hazard ratio (HR) of all-cause dementia (HR = 1.40, 95% CI: 1.11–1.77) and vascular and mixed dementia (HR = 1.88, 95% CI: 1.09–3.23), but not Alzheimer disease (HR = 1.33, 95% CI: 0.92–1.94). Among people with either prevalent or incident AF, use of anticoagulant drugs, but not antiplatelet treatment, was associated with a 60% decreased risk of dementia (HR = 0.40, 95% CI: 0.18–0.92).

**Conclusion:**

AF is associated with a faster global cognitive decline and an increased risk of dementia in older people. Use of anticoagulant drugs may reduce dementia risk in patients with AF.

Atrial fibrillation (AF) and dementia are both increasingly common as people age, exerting a heavy burden on health care systems in the aging society.^1,2^ Growing evidence has suggested that AF may have an important role in cognitive dysfunction.^3,4^ Yet, current evidence for the association of AF with cognitive decline and dementia appears to be consistent only among patients with stroke and younger-old adults,^3–6^ but not in very old people (e.g., aged 75 years and older).^7,8^ Discrepancies in previous findings could be attributable to methodologic variations; notably, incident AF has rarely been taken into consideration when relating AF to dementia and cognitive decline.^5,9,10^ This is important since the association between prevalent AF and dementia may be underestimated by survival effect and later diagnosis of AF. Furthermore, given the preventive effect of anticoagulation on AF-related stroke,^11^ it is plausible that use of anticoagulants in patients with AF may also benefit cognitive function and delay dementia onset.^12^ However, so far, there is only one retrospective register-based study from Sweden that suggested a lower risk of dementia associated with anticoagulant treatment in patients with AF.^13^ Evidence regarding the cognitive benefit of antithrombotic drugs from longitudinal population-based studies is still lacking.^14^

Therefore, using data from the population-based Swedish National Study on Aging and Care in Kungsholmen (SNAC-K), Stockholm, we sought to (1) examine the associations of AF with dementia, dementia subtypes, and cognitive decline in people aged 60 years and older, taking into account prevalent and incident AF; and (2) explore the cognitive benefit of antithrombotic treatment in patients with AF.

## Methods

### Study population

Data were derived from the SNAC-K study of people who were aged 60 years and older and living at home or in institutions in Kungsholmen, a district in central Stockholm. The study population consisted of random samples from 11 age cohorts (60, 66, 72, 78, 81, 84, 87, 90, 93, 96, and ≥99 years).^15^ Of all the 5,111 persons invited, 4,590 were alive and eligible, and 3,363 (73.3%) finally attended the baseline examination conducted between March 2001 and June 2004. We performed follow-up assessments for age cohorts younger than 78 years after 6 years (2007–2010) and every 3 years for age cohorts 78 years or older (2004–2007, 2007–2010, and 2010–2013). Of the 3,363 participants, 310 persons were diagnosed with dementia at baseline and 368 refused follow-up examinations or were no longer contactable. Thus, the analytical sample for exploring the association between AF and incidence of dementia consisted of 2,685 baseline dementia-free people. For the analysis of AF in association with cognitive decline, we further excluded 53 people who had Mini-Mental State Examination (MMSE) score <24 or missing MMSE score at baseline, and 369 people who died before the first follow-up assessment, leaving a total of 2,263 participants in the analytical sample.

### Standard protocol approvals, registrations, and patient consents

All parts of the SNAC-K (including linkage with patient register and death certificate) were approved by the regional ethical review board in Stockholm. Written informed consent was obtained from all participants or, in case of persons with cognitive impairment, from proxies (next of kin or guardians).

### Data collection

Data were gathered at baseline and each follow-up through face-to-face interviews, clinical examination, and laboratory test by trained staff following standard procedures.^16^ In brief, we collected data on demographic factors (age, sex, and education), lifestyle factors (e.g., smoking and alcohol consumption), health conditions (e.g., AF, heart failure, hypertension, and diabetes), and the use of medications. Height and weight were measured in light clothes with no shoes. Body mass index (BMI) was calculated as weight (kilograms) divided by height (meters) squared. Arterial blood pressure was measured twice at a 5-minute interval in a sitting position on the left arm with a sphygmomanometer, and the mean of the 2 readings was used in the analysis. Peripheral blood samples were taken, and *APOE* genotype, total cholesterol, and glycated hemoglobin were measured at the university's laboratory. *APOE* genotyping was performed using MALDI-TOF (matrix-assisted laser desorption/ionization time-of-flight) analysis on a modified Sequenom MassARRAY platform. During each study visit, physicians performed a comprehensive review of the participants' disease history and health conditions based on the clinical examinations, health records, self-reports, and information gathered from proxies. Diseases identified by the examining physicians were recorded using ICD-10 codes. The Swedish national inpatient register and Stockholm county inpatient and outpatient register were also linked to the SNAC-K dataset to identify participants' health conditions before baseline examination as well as during follow-ups. Information on use of medications at the baseline as well as the first and second follow-up examinations was collected by the examining physicians and computerized. All medications were coded according to the Anatomical Therapeutic Chemical (ATC) Classification System.

### Ascertainment of AF

AF was ascertained through ECG and physician's diagnosis (ICD-10 code I48) at baseline and each follow-up. In addition, patient register records (I48) for all participants were also used to detect the presence and onset date of AF prior to baseline examination and during the follow-up periods. We considered all participants with AF at baseline to have prevalent AF, whereas those who developed AF during the follow-up before the diagnosis of dementia or death were considered to have incident AF. The onset date of incident AF was defined as the date of first occurrence of symptoms identified either by the examining physician during study visits or through register records, whichever came first. Once AF was identified, participants were considered to have a history of AF through the end of follow-up.

### Assessment of global cognitive function and dementia

At each study examination, global cognitive function was assessed using MMSE. Dementia was clinically diagnosed at each study visit according to DSM-IV criteria following a validated 3-step procedure.^17^ Briefly, the examining physician made a preliminary diagnosis of dementia based on interviews, clinical examination, and cognitive testing; then a reviewing physician independently made a second preliminary diagnosis. A third opinion from a senior physician was sought in case of disagreement between the 2 preliminary diagnoses. Vascular dementia and Alzheimer disease (AD) were diagnosed according to the NINDS-AIREN (National Institute of Neurological Disorders and Stroke and Association Internationale pour la Recherché et l'Enseignement en Neurosciences) criteria^18^ and the NINCDS-ADRDA (National Institute of Neurological and Communicative Disorders and Stroke and the Alzheimer's Disease and Related Disorders Association) criteria,^19^ respectively. Since cerebrovascular and neurodegenerative pathologies often coexist in AD,^20^ participants who had a diagnosis of both vascular dementia and AD at each follow-up were considered to have mixed dementia in this study. Among people who died before the subsequent follow-up examination, physicians conducted an extensive review of their health records and death certificates to determine whether or not the participants died with dementia.

### Assessment of covariates

Education was assessed as total years of formal schooling and categorized into elementary (<8 years) and above elementary school (≥8 years). Excessive alcohol consumption was defined as having a score ≥8 using the Alcohol Use Disorders Identification Test questionnaire performed by nurses. Smoking was categorized as ever (current/former) and never smoking. Physical activity was divided into inactive (light and/or intensive exercise ≤2–3 times per month) and moderate/intensive (light, moderate, or intensive exercise several times per week). Hypertension was defined as blood pressure ≥140/90 mm Hg or current use of antihypertensive agents (ATC codes: C02, C03, C07, C08, and C09). Diabetes was defined as having a diagnosis from the physicians or register records (ICD-10 codes: E10–E14), using diabetes drugs (ATC code: A10), or having glycated hemoglobin >6.4%. High cholesterol was defined as having nonfasting total serum cholesterol ≥6.22 mmol/L or use of lipid-lowering drugs (ATC: code C10). Heart failure (ICD-10 codes: I110, I130, I132, I27, I280, I42, I43, I50, I515, I517, I528, Z941, and Z943), ischemic stroke/TIA (G45, I63–I64, and I74), and major bleeding events (both gastrointestinal and intracranial) (I60–I62, I850, I983, K26–K28, K625, K922, and D62) were identified through physician's diagnosis and patient registers.^21^ Coronary heart disease was defined as having a diagnosis from the physicians or register records (ICD-10 codes: I20–22, I24–25, Z951, and Z955), or use of nitrates (ATC code: C01DA) or ranolazine (C01EB18). Abnormal kidney function was defined as having a diagnosis from the physicians or register records (ICD 10 codes: N17–N19, I12, and I13) or having an estimated glomerular filtration rate <60 mL/min/1.73 m^2^, which was calculated based on the serum creatinine level using the Chronic Kidney Disease–Epidemiology Collaboration formula.^22^ *APOE* gene was dichotomized into carrying any ε4 allele vs no ε4 allele. Antithrombotic agents included anticoagulant drugs (ATC code: B01AA) and antiplatelet drugs (B01AC).

### Statistical analysis

Characteristics of study participants with and without prevalent AF at baseline were compared using χ^2^ tests for categorical variables and *t* tests for continuous variables. We used linear mixed-effects models to examine the association of AF with annual average changes in MMSE score over time. We used Cox proportional hazards regression models to assess the association between AF and risk of incident dementia, where follow-up time was used as the time scale. The follow-up time was estimated from the date of baseline examination to the date of dementia diagnosis, death, or the end of study period, whichever came first. To eliminate the effect of stroke/TIA in the association between AF and dementia, we repeated the analysis by excluding persons with prevalent stroke/TIA at baseline and additionally censoring persons at the dates when incident stroke/TIA occurred during the follow-up periods. We also examined the associations of AF with subtypes of dementia (incident AD, vascular dementia, and mixed dementia). Because of relatively few cases of vascular dementia (n = 37) and mixed dementia (n = 31), they were combined in the analysis of dementia subtypes; this was also in accordance with our hypothesis that AF might affect cognitive outcomes through vascular pathways. We first entered prevalent AF into the model, then AF was treated as a time-varying variable, taking into account both prevalent and incident AF. We reported results from 2 models: model 1 was adjusted for age, sex, and education, and model 2 was additionally adjusted for baseline smoking, excessive alcohol consumption, physical inactivity, BMI, diabetes, hypertension, high cholesterol, coronary heart disease, and heart failure.

Among participants with either prevalent or incident AF but without dementia at baseline or at the time when incident AF occurred, we explored the association of antithrombotic drug use with dementia risk using propensity score–weighted Cox regression models. This analysis only included data from baseline to the second follow-up (2001–2004 to 2007–2010), since information on use of medications was only available during this period. The propensity score weighting approach was adopted in order to balance the characteristics that could differ between drug users and nonusers (i.e., baseline age, sex, education, excessive alcohol consumption, diabetes, hypertension, MMSE score, coronary heart disease, heart failure, abnormal kidney function, prevalent and incident stroke/TIA, and prevalent and incident major bleeding events).^23^ We first performed logistic regressions to estimate the probabilities of using anticoagulant or antiplatelet drugs as a function of these characteristics. Then, using the predicted probabilities, a propensity score was calculated for each participant and incorporated into the Cox regression models. Additional confounders (i.e., smoking, physical inactivity, high cholesterol, and BMI) that were not included in the propensity score were further adjusted for in the models. In all the analyses, use of anticoagulant drugs and use of antiplatelet agents were treated as time-varying variables, taking into account use of the drug at baseline as well as during the follow-ups. Using the population attributable risk, we also estimated the proportion of dementia cases that can be hypothetically prevented if all patients with AF had been in the treatment group.

Missing data on covariates (<5%) were imputed using multiple imputations. We used Stata Statistical Software: Release 13 (StataCorp LP, College Station, TX) for all analyses.

### Data availability

Data on which this study is based are derived from the longitudinal population-based SNAC-K project (snac-k.se). Access to these anonymized SNAC-K data will be available upon approval by the SNAC-K data management and maintenance committee at the Aging Research Center, Karolinska Institutet, Stockholm, Sweden.

## Results

The mean age of the 2,685 participants at baseline was 73.1 (SD 10.5) years; 62.9% were women. At baseline, 243 participants (9.1%) were identified as having AF. Participants with AF were older, less educated, more likely to be men and physically inactive, and more frequently had morbidities such as hypertension, diabetes, heart failure, ischemic stroke/TIA, and coronary heart disease compared to those without prevalent AF (table 1). During the 9-year follow-up period (from 2001–2004 to 2010–2013, mean per person 5.8 years, SD 2.2 years), 279 (11.4%) of the 2,442 AF-free participants developed incident AF. A total of 399 (14.9%) incident dementia cases were detected, including 166 (6.2%) with AD and 68 (2.5%) with either vascular dementia or mixed dementia.

**Table 1:**
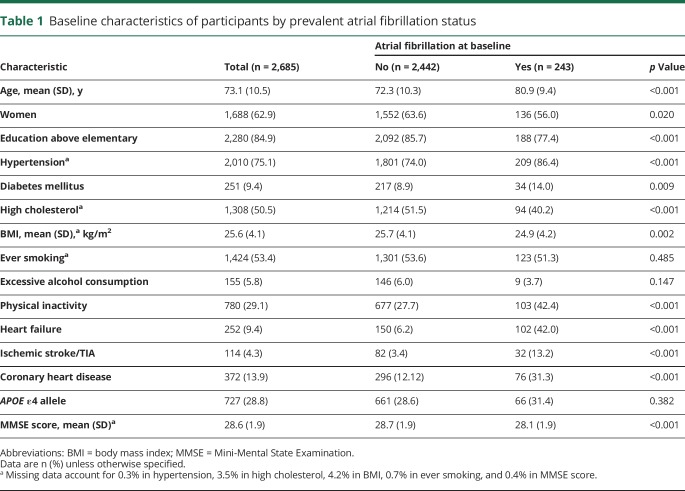
Baseline characteristics of participants by prevalent atrial fibrillation status

Linear mixed-effects models showed that AF, as a time-varying variable, was significantly associated with an accelerated average annual decline in MMSE score after adjusting for demographic factors, lifestyle factors, and chronic diseases (β coefficient = −0.24, 95% confidence interval [CI]: −0.31 to −0.16) (table 2). Cox regression models showed that AF, as a time-varying variable, was significantly associated with an increased risk of dementia after adjusting for demographics, lifestyle factors, and chronic diseases (hazard ratio [HR] = 1.40, 95% CI: 1.11–1.77), and this association was statistically evident only among women (HR = 1.46, 95% CI: 1.10–2.94) and *APOE* ε4 carriers (HR = 1.74, 95% CI: 1.17–2.59) (table 3). Excluding people with prevalent stroke/TIA at baseline (n = 114) and censoring people at the dates when incident stroke/TIA occurred during the follow-up period (n = 72) did not substantially change the results (table 3).

**Table 2:**
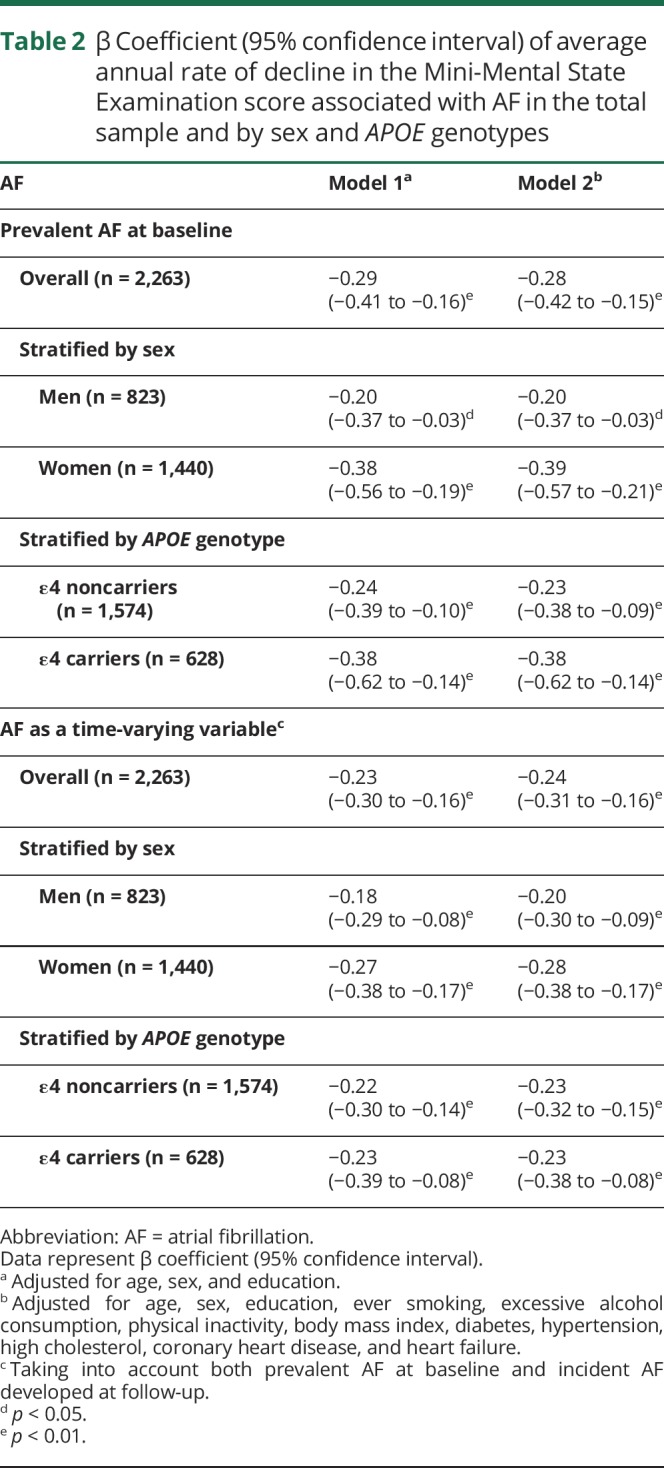
β Coefficient (95% confidence interval) of average annual rate of decline in the Mini-Mental State Examination score associated with AF in the total sample and by sex and *APOE* genotypes

**Table 3:**
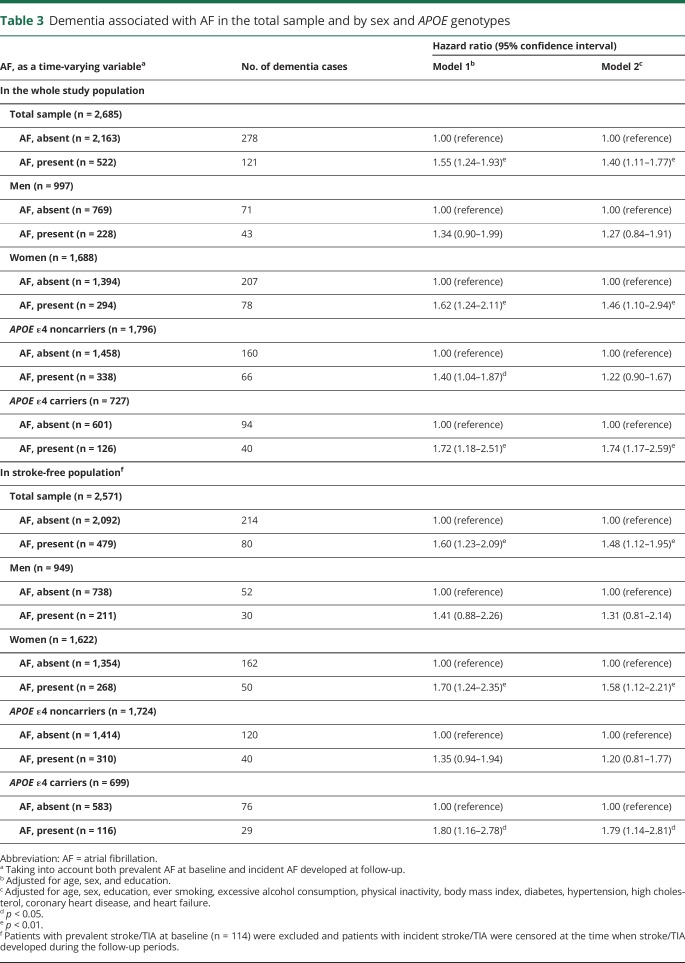
Dementia associated with AF in the total sample and by sex and *APOE* genotypes

Regarding subtypes of dementia, Cox regression analysis suggested that AF, as a time-varying variable, was significantly associated with an increased risk of the combined vascular and mixed dementia (HR = 1.88, 95% CI: 1.09–3.23) but was not significantly associated with the risk of AD (HR = 1.33, 95% CI: 0.92–1.94) (table 4).

**Table 4:**
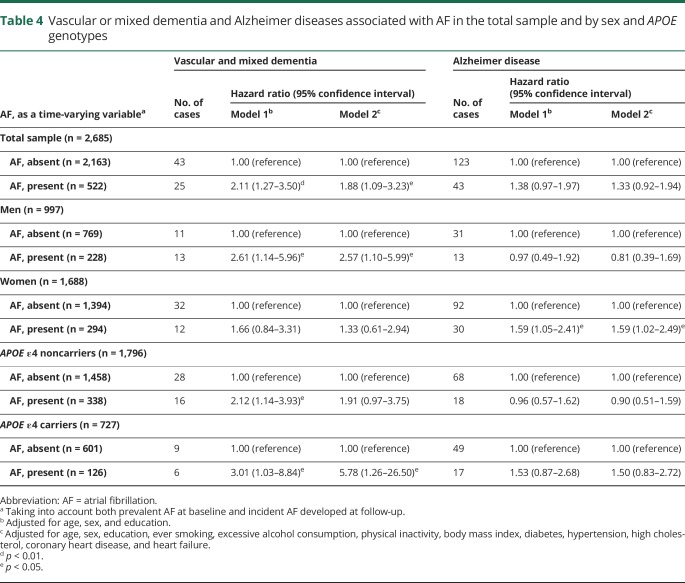
Vascular or mixed dementia and Alzheimer diseases associated with AF in the total sample and by sex and *APOE* genotypes

Among participants who had either prevalent AF at baseline or incident AF that developed during the 6-year follow-up period, propensity score–weighted Cox regression models showed that use of anticoagulant drugs was associated with a reduced risk of dementia (HR = 0.40, 95% CI: 0.18–0.92), while use of antiplatelet drugs was not significantly associated with an increased dementia risk (HR = 1.84, 95% CI: 0.99–3.42) (table 5). Assuming that there was a causal relationship between use of anticoagulant drugs and a reduced risk of dementia, we estimated that approximately 54% of the dementia cases (population attributable risk = 0.46, 95% CI: 0.22–0.95) would have been hypothetically prevented if all patients with AF had received anticoagulant drugs.

**Table 5:**
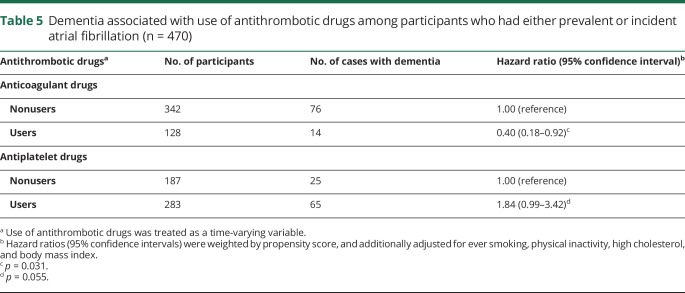
Dementia associated with use of antithrombotic drugs among participants who had either prevalent or incident atrial fibrillation (n = 470)

## Discussion

In this Swedish population-based study of people aged 60 years and older, we found that AF is associated with a faster decline in global cognitive function and an increased risk of dementia, especially vascular and mixed dementia. The role of AF in cognitive decline and dementia has been investigated in a number of population-based studies where the association appears to be more evident in middle-aged and young-old populations.^5,10,24–26^ For instance, the Atherosclerosis Risk in Communities Study^6^ (mean age 56.9 years) and the Whitehall II study^25^ (mean age 55 years) have recently reported that AF is associated with accelerated cognitive decline and higher risk of dementia. In the US register-based study, AF is associated with dementia only in people younger than 70 years.^26^ In older adults, results are mixed, whereby some studies support an association^9,27,28^ while others do not.^7,8,29^ In addition, one study specifically looking at dementia risk associated with midlife AF by *APOE* ε4 status showed that, contrary to our findings, the association is only evident among noncarriers of the *APOE* ε4 allele.^24^ Inconsistency in findings of previous studies can be partly explained by methodologic variations, such as differences in demographic features of study population, length of follow-up time, sample size, and attrition rate. In addition, incident AF was rarely accounted for in studies involving older populations, which could have underestimated the association of AF with dementia since diagnosis of AF in the general population is often delayed because of the temporal pattern of AF episodes.^30^

The association of AF with cognitive decline and dementia could be explained by vascular pathways such as clinical and silent cerebrovascular lesions due to thromboembolism associated with blood stasis and systemic hypercoagulable state, and cerebral hypoperfusion associated with low cardiac output in AF.^31–33^ Indeed, our study showed a rather strong association of AF with the combined vascular and mixed dementia, but not with AD. A few studies have investigated the association between AF and dementia subtypes, which yielded mixed results; some studies reported a higher risk of vascular dementia related to AF,^26,34^ while others supported an elevated risk mainly for AD.^28,35^ However, most of these previous studies did not consider mixed dementia cases caused by both vascular and AD pathologies in the brain. Indeed, population-based neuroimaging and neuropathologic studies have demonstrated that in most cases of clinically diagnosed dementia, cerebrovascular and neurodegenerative features coexist.^36^ Current view suggests that in elderly people with AD pathologies insufficient to reach the threshold of dementia, the additional presence of vascular brain injuries may hasten the expression of dementia syndromes.^20,36,37^ In line with this view, our findings have shown that AF is associated with a 6-fold elevated risk of combined vascular and mixed dementia among *APOE* ε4 carriers, while the association is not evident among noncarriers. This indicates that while AF itself might not be enough to cause clinical dementia, the coexistence of AD pathology associated with *APOE* ε4 allele and cerebrovascular lesions related to AF could lead to a substantial risk of dementia than either process alone. It is also plausible that cerebral ischemia and hypoperfusion caused by AF could induce the deposition of β-amyloid precursor protein, amyloidogenic fragments, and tau-like pathology associated with AD, which additively or synergistically contributes to a faster cognitive decline and an earlier onset of dementia.^36,37^

The finding of an association between AF and an increased risk of dementia and cognitive decline has potential therapeutic implications. However, very few population-based studies have evaluated the cognitive consequences of antithrombotic treatment in older patients with AF.^10,13,38^ One randomized controlled trial that assessed cognitive outcomes in relation to antithrombotic treatment in patients with AF (aged 75 years and older) found no evidence that anticoagulant drugs were superior to antiplatelet drugs in reducing the risk of global cognitive decline.^39^ The retrospective analysis of data from the patient and drug registers in Sweden indicated that risk of dementia was higher in people with AF but without anticoagulant treatment than those on treatment.^13^ Our population-based study showed that use of anticoagulant drugs, but not antiplatelet treatment, was associated with a reduced risk of dementia in patients with AF. This is in agreement with the 2012 European Society of Cardiology guidelines, which recommend anticoagulants as the first-line treatment in patients with AF while the use of antiplatelet therapy should be limited.^11^ Assuming that there is a causal relationship between use of anticoagulant drugs and a reduced risk of dementia, we estimated that approximately 54% of dementia cases would have been prevented if all patients with AF had used anticoagulant drugs. Our findings shed light on the added cognitive benefit of anticoagulant drugs in patients with AF apart from stroke prevention. Additional efforts should be made to improve anticoagulant treatment in elderly patients with AF, since anticoagulation remains underprescribed, particularly in older patients with AF.^40,41^ Furthermore, in the era of novel oral anticoagulants, which have lower risk of hemorrhage and do not require serial monitoring, future studies are needed to assess the net cognitive benefit of novel oral anticoagulants.^33^

Our study has several limitations. First, we could not distinguish subtypes of AF (e.g., paroxysmal, persistent, and permanent). Although we used multiple sources to identify AF cases, we still could have missed asymptomatic AF cases. Second, the MMSE is a screening test for global cognitive dysfunction that may not be sensitive to subtle changes in certain cognitive domains (e.g., executive and visuospatial function), especially among highly educated individuals, which could have led to an underestimation of the association between AF and cognitive decline. Third, lack of data on the duration of antithrombotic treatment and the quality of anticoagulation control (i.e., international normalized ratio) represents another limitation of this study, as evidence has shown that poor quality of anticoagulant control (i.e., under- or overanticoagulation) might be a risk factor for dementia and that low percentage of time in therapeutic range was associated with an increased dementia risk among patients with AF.^42^ However, this might only have limited effect on our results, as a study of the Swedish national quality registry for AF and anticoagulation showed that time in therapeutic range for patients on anticoagulant therapy was generally good in Sweden (76.2%).^43^ Fourth, although we used the propensity score and additionally controlled for multiple variables in our analysis to minimize the indication and confounding bias, residual confounding might still exist because of imperfect measurements of those factors and other unmeasured factors (e.g., cerebral amyloid angiopathy), and physicians or geriatricians might also be more cautious in prescribing anticoagulant drugs to older patients with cognitive difficulties but without a diagnosis of dementia. Finally, the SNAC-K population is fairly homogeneous, consisting mostly of highly educated Caucasians from an urban district. This needs to be kept in mind when generalizing our findings to other populations.

Taken together, this Swedish longitudinal population-based study suggests that AF is associated with an increased risk of dementia and a faster decline in global cognitive function in older people. We also found evidence suggesting that use of anticoagulant drugs may prevent older patients with AF from developing dementia. Future studies are warranted to clarify the possible cognitive benefits of anticoagulant drugs in patients with AF.

## Publication history

Received by *Neurology* January 19, 2018. Accepted in final form July 23, 2018.

## Glossary

AD: Alzheimer disease
AF: atrial fibrillation
ATC: Anatomical Therapeutic Chemical
BMI: body mass index
CI: confidence interval
DSM-IV: *Diagnostic and Statistical Manual of Mental Disorders* (Fourth Edition)
HR: hazard ratio
ICD-10: *International Classification of Diseases, Tenth Revision*
MMSE: Mini-Mental State Examination
SNAC-K: Swedish National Study on Aging and Care in Kungsholmen
